# ﻿Contribution to the knowledge of the genus *Solskyia* Solsky, 1881 (Coleoptera, Tenebrionidae, Akidini) from China

**DOI:** 10.3897/zookeys.1122.86071

**Published:** 2022-09-19

**Authors:** Xing-Long Bai, Jing-Ze Liu, Guo-Dong Ren

**Affiliations:** 1 Hebei Key Laboratory of Animal Physiology, Biochemistry and Molecular Biology, College of Life Sciences, Hebei Normal University, Shijiazhuang, Hebei 050024, China Hebei Normal University Shijiazhuang China; 2 The Key Laboratory of Zoological Systematics and Application, School of Life Sciences, Institute of Life Science and Green Development, Hebei University, Baoding, Hebei 071002, China Hebei University Baoding China

**Keywords:** Biology, darkling beetles, ecology, larvae, new species, Pimeliinae, redescription

## Abstract

Two new species of the genus *Solskyia*, *S.infossata***sp. nov.** and *S.lhozhaga***sp. nov.**, are described and illustrated from Xizang, China. *Solskyialhasana* is redescribed and figured based on a male, and new material of *S.caporiaccoi* and *S.parvicollis* from China is documented. The ecology and biology of adults and larvae is briefly introduced. Furthermore, photographs of habitat, and a key to Chinese species are presented.

## ﻿Introduction

The tribe Akidini Billberg (Tenebrionidae, Pimeliinae) is divided into five genera: *Akis* Herbst, *Cyphogenia* Solier, *Morica* Dejean, *Sarothropus* Kraatz, and *Solskyia* Solsky (Bouchard et al. 2021). Two of them, *Cyphogenia* and *Solskyia*, are recorded from China (Iwan et al. 2020). *Cyphogenia* can be easily distinguished from *Solskyia* by the onychium, which is lobed ventrally.

*Solskia* used by Semenov (1891) and *Solskya* used by Semenov (1908) turned out to be incorrect subsequent spellings for *Solskyia* Solsky, 1881 (Bouchard et al. 2021), which have been followed by subsequent authors (e.g., Kaszab 1965; Ren and Yu 2000). To date, twelve species have been described in the genus *Solskyia* worldwide. Species of this genus are known to occur only in Asia, distributed from Central Asia to Kashmir and the Himalayas, and also found in China (Iwan et al. 2020). Most of them were mentioned in publications or monographs on tenebrionid beetles (e.g., Kraatz 1865; Reitter 1904; Español 1961; Medvedev 1968; Ren and Yu 2000), scientific reports of expeditions (e.g., Bates 1879; Della Beffa 1931; Gridelli 1934), and studies of regional faunas (e.g., Solsky 1881; Semenov 1889, 1891; Fairmaire 1891; Kaszab 1965, 1970). Among them, the contribution of Español (1961) was very valuable, including known previous species of the genus; two known species (i.e., *S.grombczewskii* Semenov, *S.parvicollis* Kraatz) were redescribed and two new species (i.e., *S.kaszabi* Español, *S.schmidi* Español) were described with line drawings of the habitus and male genitalia; an identification key to all known species of the genus was presented.

Three species of the genus *Solskyia* have been recorded from Xinjiang and Xizang, China until now (Ren and Yu 2000; Iwan et al. 2020). The first species, *Akisparvicollis* Kraatz, 1865, was mentioned in the publication of Kraatz’s revision of the Old World tenebrionids [Kraatz (1865); from Lacordaire’s group of Akisides]. The second species, *Solskyiacaporiaccoi* Gridelli, 1934 was collected by the Italian expedition to Karakoram. The last species, *Solskyialhasana* Ren & Yu, 2000, was described from Xizang, China based on a single female specimen.

This study aims to present an overview of the species belonging to the genus *Solskyia* in China, with a redescription of *S.lhasana* based on a male and the description of two new species from Xizang.

## ﻿Materials and methods

The specimens were examined and dissected under a Nikon SMZ800 microscope, and photographs were taken using Canon EOS 5DSR camera. Aedeagi and ovipositors were detached from the body with insect pins, then glued to separate cards and pinned under the specimens. Specimens examined in this study are deposited in **HBUM** (Hebei University Museum, Baoding, China), and **IPPP** (Collection of Insect Prevention of The Potala Palace [布达拉宫防虫标本室], Lhasa, China). Larvae mentioned in this paper were collected along with adults in the same localities at the same time, with confirmed identity by emerging into adults by rearing in the laboratory. A single slash (/) separates data of different lines on a label, a double slash (//) separates data of different labels, authors’ remarks are enclosed in brackets “ []”.

### ﻿Ecology and biology

Species of *Solskyia* live in the semi-deserts and mountains of Asia (Figs 15, 19, 23, 26, 30). Generational overlap is present in *Solskyia* species, and adults and larvae can be found at the same time. Generally, they are hidden under stones, in crevices and caves (Figs 16, 23, 27, 30) during the day. In contrast, they are more frequent during the night, and can be found on the ground (Fig. 21), representing a large part of the local darkling beetle species abundance. Larvae can dig (Fig. 18). Adults secrete fluid from mouthparts when startled (Fig. 22), possibly as a form of defense.

A larva was collected from Günsa Township, Gar County, Xizang in August 24, 2015, and brought back to the laboratory for rearing. Eclosion into an adult occurred in late May 2016. The pupal stage was very short, just a few days.

## ﻿Taxonomy

### ﻿Genus Solskyia

Taxon classificationAnimaliaColeopteraTenebrionidae

Solsky, 1881

A49E86F9-5709-5D6F-BA79-C8BF159F99CE

Solskyia Solsky, 1881: 48; Gridelli 1934: 47; Español 1961: 123; Ren and Yu 2000: 325 (incorrect spelling as Solskia); Löbl et al. 2008: 127; Iwan et al. 2020: 138.

#### Type species.

*Solskyiaperegrina* Solsky, 1881, by monotypy.

### ﻿ Solskyia caporiaccoi

Taxon classificationAnimaliaColeopteraTenebrionidae

Gridelli, 1934

211805CE-EA68-524E-A1DC-5A3C634340F2

Figs 15–18 , 19–22

Solskyia caporiaccoi Gridelli, 1934: 53; Español 1961: 131; Ren and Yu 2000: 326 (incorrect spelling as Solskia); Löbl et al. 2008: 127; Iwan et al. 2020: 138.

#### Material examined.

**China**: 1♀ (HBUM), Burang County, Xizang, 1974-VIII-18, leg. Ji-Jun Li; 1♀ (HBUM), Burang County, Xizang, 2006-VIII-20, leg. Ming-Sheng Zhu; 1 ex. (HBUM), Burang County, Xizang, 30°17'11.8"N, 81°10'30.6"E, 3875 m, 2022-VII-8, leg. Jun-Sheng Shan; 3 ex. (HBUM), Burang County, Xizang, 30°16.5852'N, 81°11.4735'E, 4006 m, 2022-VII-10, leg. Guo-Dong Ren, Yi-Ping Niu, Xing-Long Bai, Kai-Xuan Liu; 2 ex. (HBUM), Qangzê Township, Zanda County, Xizang, 31°41.282'N, 79°46.610'E, 4420 m, 2015-VIII-24, leg. Guo-Dong Ren, Xing-Long Bai, Jun-Sheng Shan; 22 ex. (HBUM), Qangzê Township, Zanda County, Xizang, 31°49.779'N, 79°37.540'E, Alt. 4222 m, 2018-VIII-11, leg. Xing-Long Bai, Zi-Yuan Hu, Ming-Min Ma; 2 ex. (HBUM), Zanda Tulin, Zanda County, Xizang, 31°40.548'N, 79°44.382'E, Alt. 4047 m, 2018-VIII-11, leg. Xing-Long Bai, Zi-Yuan Hu, Ming-Min Ma; 1 ex. (HBUM), Zanda Tulin, Zanda County, Xizang, 31°33.846'N, 79°50.181'E, Alt. 4129 m, 2018-VIII-13, leg. Xing-Long Bai, Zi-Yuan Hu, Ming-Min Ma; 9 ex. (HBUM), Diyag Township, Zanda County, Xizang, 31°48.077'N, 78°50.666'E, Alt. 2956 m, 2018-VIII-12, leg. Xing-Long Bai, Zi-Yuan Hu, Ming-Min Ma; 61 ex. (HBUM), Diyag Township, Zanda County, Xizang, 31°47.026'N, 78°52.052'E, Alt. 2978 m, 2018-VIII-12, leg. Xing-Long Bai, Zi-Yuan Hu, Ming-Min Ma.

#### Distribution.

China: Xizang; Kashmir.

### ﻿ Solskyia infossata sp. nov.

Taxon classificationAnimaliaColeopteraTenebrionidae

26C2B49B-9116-5C23-85F1-77C24EE76ACC

https://zoobank.org/DB08D547-1250-4800-B9CE-73A91270A069

Figs 1 , 2–5 , 23–25

#### Type material.

***Holotype***: ♂ (HBUM), 2018-VIII-6 / 西藏 林芝市朗县 [Nang County, Nyingchi City, Xizang] / 闫霞 [leg. Xia Yan] 3016 m / 西华师大标本馆 [Museum of China West Normal University]. ***Paratypes***: 5 ex. (HBUM), 2018-VIII-6 / 西藏 林芝市朗县 [Nang County, Nyingchi City, Xizang] / 闫霞 [leg. Xia Yan] 3016 m / 西华师大标本馆 [Museum of China West Normal University]; 12 ex. (HBUM), 2019-VII-28 / 西藏加查加查镇奴巧村 [Nuqiao Village, Gyaca Town, Gyaca County, Xizang] / 潘昭 李秀敏 文明 王兰蕊 [leg. Zhao Pan, Xiu-Min Li, Ming Wen, Lan-Rui Wang] / 河北大学博物馆 [Hebei University Museum] // 29°08'19"N, 92°39'11"E / Alt. 3285 m / 河北大学博物馆 [Hebei University Museum].

**Figure 1.**
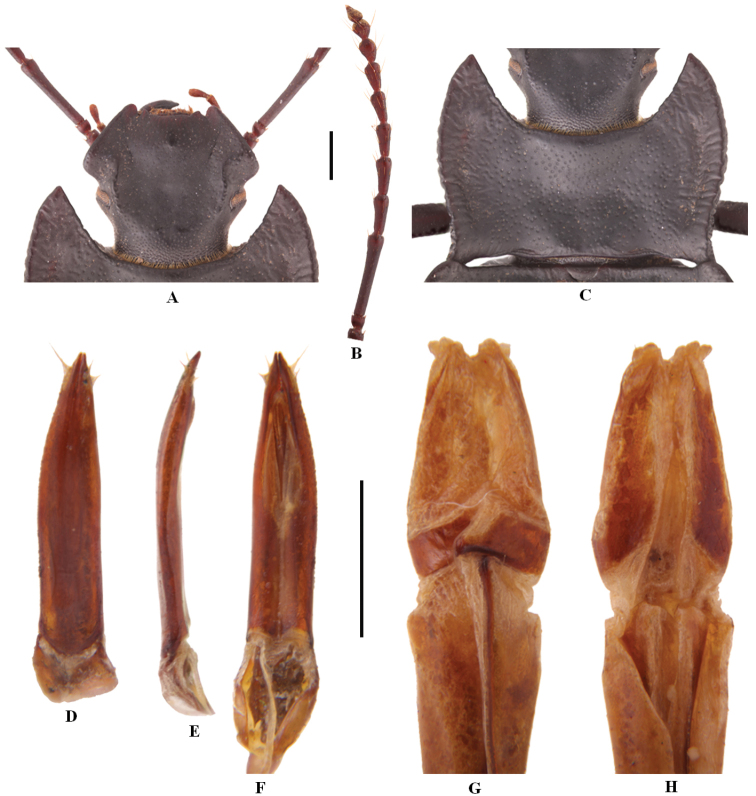
Characters of *Solskyiainfossata* sp. nov. **A–F** male **A** head **B** antenna **C** pronotum **D–F** aedeagus in dorsal, lateral and ventral view, respectively **G, H** female: ovipositor in dorsal and ventral view, respectively. Scale bars: 3.5 mm (**A–C**); 1.0 mm (**D–H**).

#### Diagnosis.

This new species closely resembles *S.lhozhaga* sp. nov., but can be distinguished from the latter by the following characters: (1) punctures on head finer (coarser in *S.lhozhaga*); (2) lateral sides of pronotum weakly “S” curved (arcuate in *S.lhozhaga*), posterior angles sharp and protruding outwards (weakly obtuse in *S.lhozhaga*). This new species is also somewhat similar to *S.lhasana*, it differs from the later by the following characters: (1) body wide-oval (elongate-oval in *S.lhasana*); (2) lateral margins of pronotum weakly “S” curved (arcuate in *S.lhasana*); (3) elytra wide and short (narrow and long in *S.lhasana*), base wider than pronotum (narrower in *S.lhasana*), lateral margins widest near middle (subparallel in *S.lhasana*), humeri rectangular-angled, rounded apically (widely obtuse in *S.lhasana*), surface of elytra and epipleura with punctures (granules in *S.lhasana*).

**Figure 2–5.**
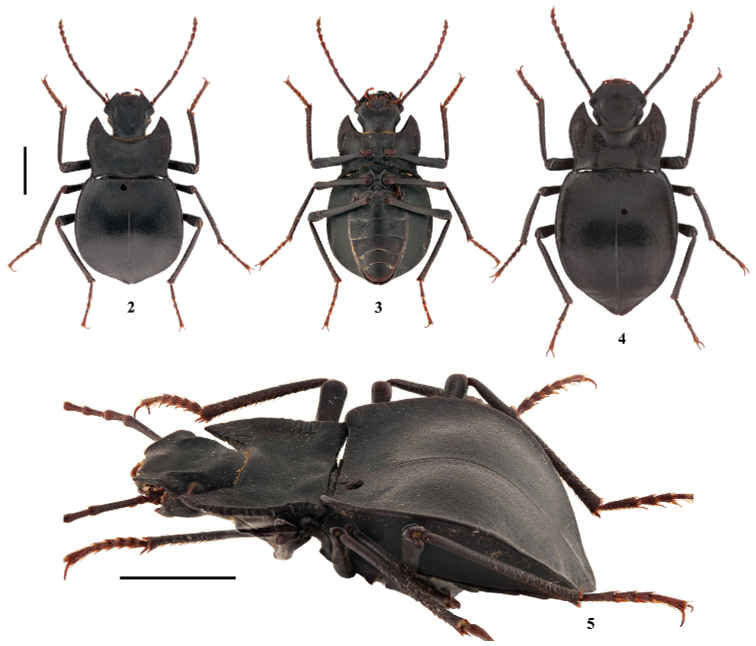
Habitus of *Solskyiainfossata* sp. nov. **2–3, 5** male (holotype) in dorsal, ventral and oblique view, respectively **4** female (paratype). Scale bars: 3.5 mm.

#### Distribution.

China: Xizang.

#### Etymology.

The species name is derived from its depressed dorsal side of the body.

#### Description.

Total length 14.6–18.1 mm; width 8.0–9.2 mm. Wide-oval, dorsal side depressed, ventral side strongly convex. Body black, weakly shiny; labrum, palpi, antennomeres IX–XI and tarsi brown.

***Head*.** Anterior margin of labrum nearly straight, with long setae, lateral margins parallel, distal part punctate, basal half smooth. Anterior margin of clypeus nearly straight at middle and serrate, lateral angles toothed and protruding forwards, with a deep incision between lateral angles and anterior part of genae; surface convex, sparsely and finely punctate. Clypeogenal suture indicated. Dorsal surface of head flat, lateral sides above eyes longitudinally carinate, shallowly, sparsely and finely punctate. Genal margins nearly right-angled protruding outwards before eyes, straightly converging forwards, strongly and arcuately narrowing backwards, sparsely and finely punctate. Eyes transverse. Temples behind eyes strongly and roundly narrowing backwards, punctures larger. Mentum transverse, anterior margin widely triangular emarginate, lateral margins subparallel and tilt up. Antennae slender and long, reaching beyond the pronotal base; basal part of antennomere I invisible in dorsal view, II very short, III very long, IV–VIII gradually shorter, X nearly spherical; XI sharped-oval, narrow and small, closely joint with X; III–VIII thicker at apex; apex of I–X with sparse setae and gradually longer; inner side of apex of VIII, inner side and outside of apex of IX–X, apex of XI with sensilla.

***Prothorax*.** Pronotum transverse, subcordiform, widest at middle, 2.4 times as wide as long, significantly wider than head; anterior margin deeply emarginate, beaded laterally; lateral margins weakly “S” curved, broadly beaded and strongly tilt up; posterior margin bisinuate, finely beaded; anterior angles sharp and protruding forwards, posterior angles sharp and protruding; lateral margins and sides wrinkled; surface strongly depressed with transverse depression in middle, weakly triangular convex in middle of anterior margin, shallowly, sparsely and coarsely punctate. Prothoracic hypomera depressed, smooth, shallowly and sparsely punctate. Prosternal process weakly sloping behind procoxae, apex blunt in lateral view.

***Pterothorax*.** Elytra wide-oval, widest near middle, 1.1 times as long as wide; anterior margin nearly straight, base slightly wider than pronotum; lateral margins arcuate, weakly narrowing toward base and strongly narrowing toward apex from middle, lateral margins raised, humeri broad and wrinkled, right-angled, rounded apically; surface depressed, more deeply at base, but strongly convex near the middle of anterior margin, declivity sharply sloping downwards; sparsely and finely punctate, shallowly near base, lateral sides and apex, shallowly and coarsely wrinkled; epipleura wide, weakly convex, surface matte, shallowly, sparsely and finely punctate, shallowly and coarsely wrinkled. Scutellum triangular.

***Abdomen*.** Ventrites strongly convex, densely and coarsely punctate, sparsely and shallowly near lateral sides and apex of the last ventrite; apical margin of the last ventrite widely rounded.

***Legs*.** Slender and long; femora claviform, smooth; tibiae straight, rough; ventral surface of pro- and mesotarsomeres I–IV and metatarsomeres I–III with hairy tuft at apex; claws well developed.

***Aedeagus*.** As in Fig. 1D–F. Length 2.6 mm, width 0.6 mm. Parameres length 2.0 mm, width 0.4 mm.

***Ovipositor*.** As in Fig. 1G, H.

***Sexual dimorphism*.** Females usually with slightly wider and more convex elytra, but in many cases, it is impossible to distinguish the two sexes without extracting the genitalia.

### ﻿ Solskyia lhasana

Taxon classificationAnimaliaColeopteraTenebrionidae

Ren & Yu, 2000

D47B4399-7444-576C-88A8-FB57BCC0E7A2

Figs 6 , 7–9

Solskyia lhasana Ren & Yu, 2000: 325 (incorrect spelling as Solskia); Löbl et al. 2008: 127; Iwan et al. 2020: 138.

#### Type material.

***Holotype***: ♀ (HBUM), 1979-V-10 / 西藏拉萨 [Lhasa, Xizang] / 李法圣 [leg. Fa-Sheng Li] / 河北大学博物馆 [Hebei University Museum] // HOLOTYPE.

**Figure 6.**
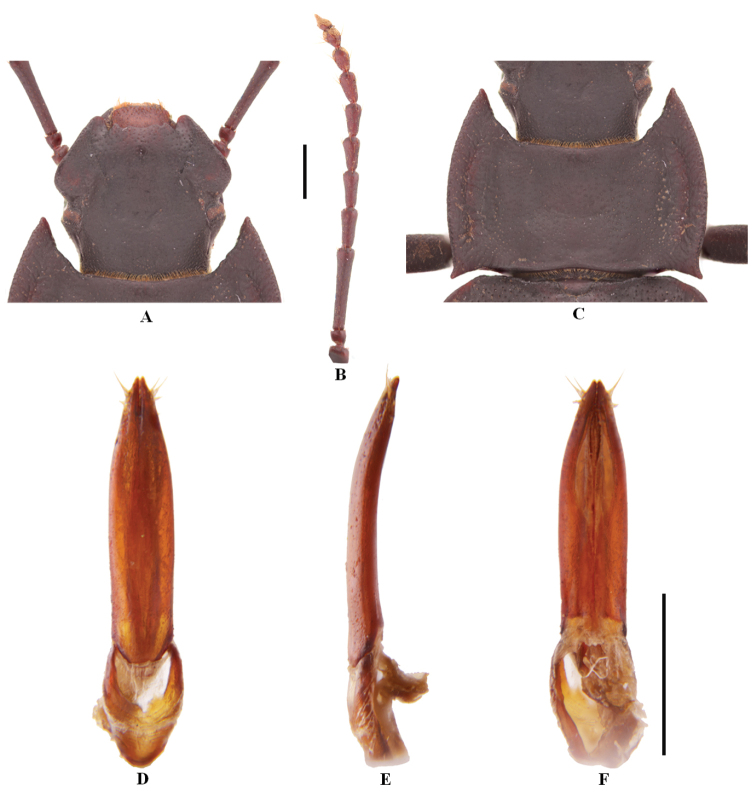
Characters of *Solskyialhasana* Ren & Yu (male) **A** head **B** antenna **C** pronotum **D–F** aedeagus in dorsal, lateral and ventral view, respectively. Scale bars: 3.5 mm (**A–C**); 1.0 mm (**D–F**).

#### Additional material.

**China**: 1♂ (HBUM), Zêtang Town, Shannan City, Xizang, 3538 m, 2018-VIII-13, leg. Liang Xiang; 1 ex. (IPPP), The Potala Palace, Lhasa, Xizang.

**Figure 7–9.**
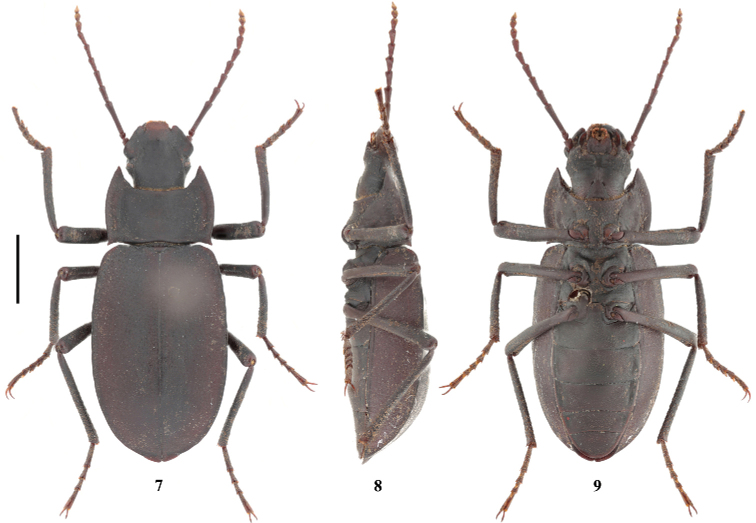
Habitus of *Solskyialhasana* Ren & Yu (male) in dorsal, lateral and ventral view, respectively. Scale bars: 3.5 mm.

#### Distribution.

China: Xizang.

#### Description of male.

Length 17.9 mm; width 7.0 mm. Oval, elongated, dorsal side depressed, ventral side strongly convex. Body black, weakly shiny; labrum, palpi, antennomeres IX–XI and tarsi brown.

***Head*.** Anterior margin of labrum nearly straight, with long setae, lateral margins parallel, distal part punctate, basal half smooth. Anterior margin of clypeus serrate, lateral angles weakly toothed and protruding forwards, with a shallow incision between lateral angles and anterior part of genae; surface convex, sparsely and finely punctate. Clypeogenal suture indicated. Dorsal surface of head flat, lateral sides above eyes longitudinally carinate, sparsely and finely punctate. Genal margins weakly obtuse-angled protruding outwards before eyes, straightly converging forwards, strongly and arcuately narrowing backwards, sparsely and finely punctate. Eyes transverse. Temples behind eyes strongly and roundly narrowing backwards, finely punctate. Mentum transverse, anterior margin widely triangularly emarginate, lateral margins arcuate and elevated. Antennae slender and long, reaching beyond the pronotal base; basal part of antennomere I invisible in dorsal view, II very short, III very long, IV–VIII gradually shorter, X nearly spherical; XI sharped-oval, narrow and small, closely joint with X; III–VIII thicker at apex; apex of I–X with sparse setae and gradually longer; inner side of apex of VIII, inner side and outside of apex of IX–X, apex of XI with sensilla.

***Prothorax*.** Pronotum transverse, widest at middle, 2.2 times as wide as long, significantly wider than head; anterior margin deeply emarginate, beaded laterally; lateral margins arcuate, broadly beaded and strongly raised; posterior margin bisinuate, finely rimmed; anterior angles sharp and protruding forwards, posterior angles sharp and protruding outwards; lateral margins and sides wrinkled; surface strongly depressed with transverse depression in middle, weakly triangular convex in middle of anterior margin, shallowly, sparsely and finely punctate. Prothoracic hypomera depressed, smooth, shallowly and sparsely punctate. Prosternal process weakly sloping behind procoxae, apex blunt in lateral view.

***Pterothorax*.** Elytra oval elongated, widest near middle, 1.6 times as long as wide; anterior margin nearly straight, base narrower than pronotum; lateral sides subparallel, weakly narrowing toward base and strongly narrowing toward apex from middle, lateral margin raised; humeri widely obtuse-angled; surface depressed, deeper at base, declivity sharply sloping downwards; sparsely and finely granulated, shallowly and coarsely wrinkled; epipleura wide, weakly convex, sparsely and finely granulated, shallowly and coarsely wrinkled. Scutellum semicircular.

***Abdomen*.** Ventrites strongly convex, shallowly punctate, gradually finer toward lateral sides and apex of the last ventrite; apical margin of the last ventrite widely rounded.

***Legs*.** Slender and long; femora claviform, smooth; tibiae straight, rough; ventral surface of pro- and mesotarsomeres I–IV and metatarsomeres I–III with hairy tuft at apex; claws well developed.

***Aedeagus*.** As in Fig. 6D–F. Length 2.4 mm, width 0.5 mm. Parameres length 1.8 mm, width 0.4 mm.

### ﻿ Solskyia lhozhaga sp. nov.

Taxon classificationAnimaliaColeopteraTenebrionidae

44B15F94-5C96-5BFA-AFB3-B191EE64026D

https://zoobank.org/72724339-6D66-44A5-AB0C-D2CA358CA483

Figs 10 , 11–14 , 26–29

#### Type material.

***Holotype***: ♂ (HBUM), 2014-VIII-7 / 西藏洛扎县生格乡 [Sênggê Township, Lhozhag County, Xizang] / 任国栋 白兴龙 单军生 [leg. Guo-Dong Ren, Xing-Long Bai, Jun-Sheng Shan] / 河北大学博物馆 [Hebei University Museum] // 28°12.752'N, 91°00.770'E / Alt. 3225 m / 河北大学博物馆 [Hebei University Museum]. ***Paratypes***: 20 ex. (HBUM), 2014-VIII-7 / 西藏洛扎县生格乡 [Sênggê Township, Lhozhag County, Xizang] / 任国栋 白兴龙 单军生 [leg. Guo-Dong Ren, Xing-Long Bai, Jun-Sheng Shan] / 河北大学博物馆 [Hebei University Museum] // 28°12.752'N, 91°00.770'E / Alt. 3225 m / 河北大学博物馆 [Hebei University Museum]; 1 ex. (HBUM), 2022-VII-22 / 西藏洛扎拉康镇杰拉山 [Gyai La Shan, Lhakang Town, Lhozhag County, Xizang] / 任国栋 牛一平 白兴龙 刘凯璇 [leg. Guo-Dong Ren, Yi-Ping Niu, Xing-Long Bai, Kai-Xuan Liu] / 河北大学博物馆 [Hebei University Museum] // 28°08.0839'N, 91°09.3682'E / Alt. 3302 m / 河北大学博物馆 [Hebei University Museum].

**Figure 10.**
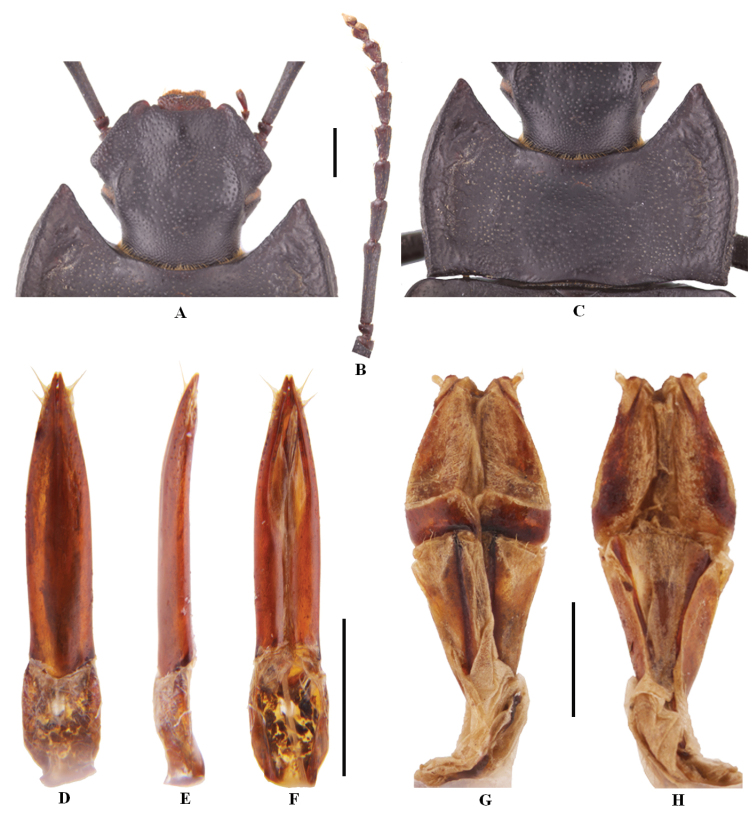
Characters of *Solskyialhozhaga* sp. nov. **A–F** male **A** head **B** antenna **C** pronotum **D–F** aedeagus in dorsal, lateral and ventral view, respectively **G, H** female: ovipositor in dorsal and ventral view, respectively. Scale bars: 3.5 mm (**A–C**); 1.0 mm (**D–H**).

#### Diagnosis.

This new species closely resembles *S.infossata* sp. nov., but can be distinguished from the latter by the following characters: (1) punctures on head coarser (finer in *S.infossata*); (2) lateral margins of pronotum arcuate (weakly “S” curved in *S.infossata*), posterior angles weakly obtuse (sharp and protruding outwards in *S.infossata*). This new species is also somewhat similar to *S.lhasana*, it differs from the later by the following characters: (1) body wide-oval (oval elongated in *S.lhasana*); (2) punctures on head coarser (finer in *S.lhasana*); (3) posterior angles of pronotum weakly obtuse (sharp and protruding outwards in *S.lhasana*), coarsely punctate (finely in *S.lhasana*); (4) elytra wide and short (narrow and long in *S.lhasana*), base wider than pronotum (narrower in *S.lhasana*), lateral margins widest near middle (subparallel in *S.lhasana*), humeri right-angled, rounded apically (widely obtuse in *S.lhasana*), surface of elytra and epipleura with punctures (granules in *S.lhasana*).

**Figure 11–14.**
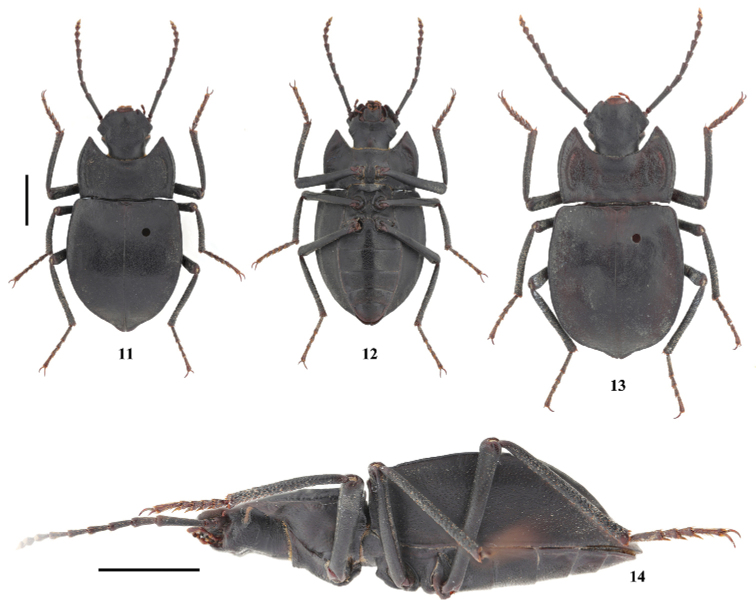
Habitus of *Solskyialhozhaga* sp. nov. **11, 12, 14** male (holotype) in dorsal, ventral and lateral view, respectively **13** female (paratype). Scale bars: 3.5 mm.

#### Distribution.

China: Xizang.

#### Etymology.

The species name is derived from the type locality – Lhozhag.

#### Description.

Total length 16.6–19.2 mm; width 8.5–9.9 mm. Wide-oval, dorsal side depressed, ventral side strongly convex. Body black, weakly shiny; labrum, palpi, antennomeres IX–XI and tarsi brown.

**Figure 15–18.**
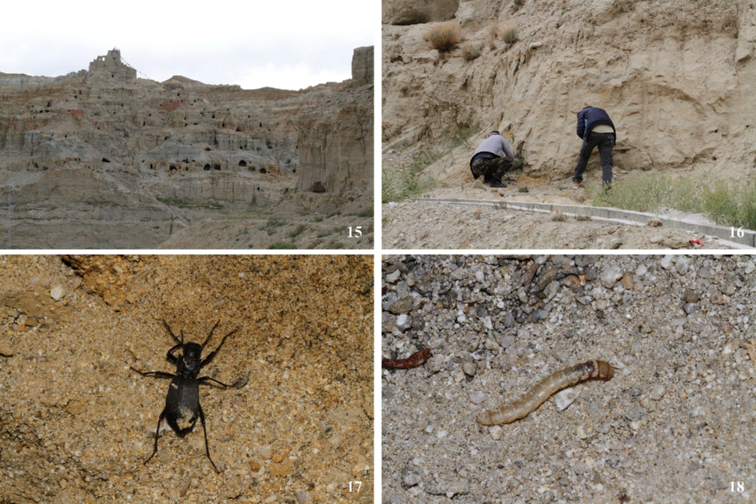
Habitat **15, 16** adult **17** and larva **18** of *Solskyiacaporiaccoi* Gridelli in Qangzê Township, Zanda County, Xizang, China.

***Head*.** Anterior margin of labrum nearly straight, with long setae, lateral margins parallel, distal part punctate, basal half smooth. Anterior margin of clypeus nearly straight at middle and serrate, lateral angles weakly toothed and protruding forwards, with a shallow incision between lateral angles and anterior part of genae; surface convex, sparsely and coarsely punctate. Clypeogenal suture inconspicuous. Dorsal surface of head flat, lateral sides above eyes longitudinally carinate, sparsely and coarsely punctate. Genal margins nearly right-angled protruding outwards before eyes, straightly converging forwards, strongly and arcuately narrowing backwards, sparsely and coarsely punctate. Eyes transverse. Temples behind eyes strongly and roundly narrowing backwards, coarsely punctate. Mentum transverse, anterior margin widely triangularly emarginate, lateral margins subparallel and raised. Antennae slender and long, reaching beyond the pronotal base; basal part of antennomere I invisible in dorsal view, II very short, III very long, IV–VIII gradually shorter, X nearly spherical; XI sharped-oval, narrow and small, closely joint with X; III–VIII thicker at apex; apex of I–X with sparse setae and gradually longer; inner side of apex of VIII, inner side and outside of apex of IX–X, apex of XI with sensilla.

**Figure 19–22.**
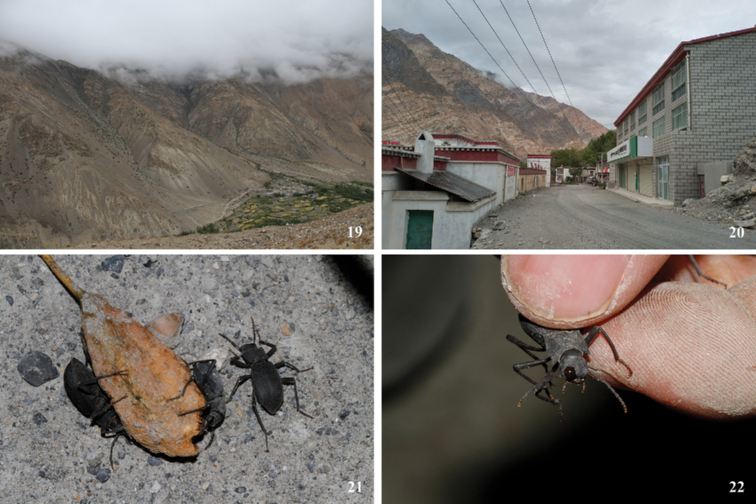
Habitat **19, 20** and adults **21, 22** of *Solskyiacaporiaccoi* Gridelli in Diyag Township, Zanda County, Xizang, China.

***Prothorax*.** Pronotum transverse, widest at middle, 2.6 times as wide as long, significantly wider than head; anterior margin deeply emarginate, beaded laterally; lateral margins arcuate, broadly beaded and strongly raised; posterior margin bisinuate, finely beaded; anterior angles sharp and protruding forwards, posterior angles weakly obtuse; lateral margins and sides wrinkled; surface strongly depressed with transverse depression in middle, weakly triangular convex in middle of anterior margin, sparsely and coarsely punctate. Prothoracic hypomera depressed, smooth, shallowly and sparsely punctate. Prosternal process weakly sloping behind procoxae, apex blunt in lateral view.

**Figure 23–25.**
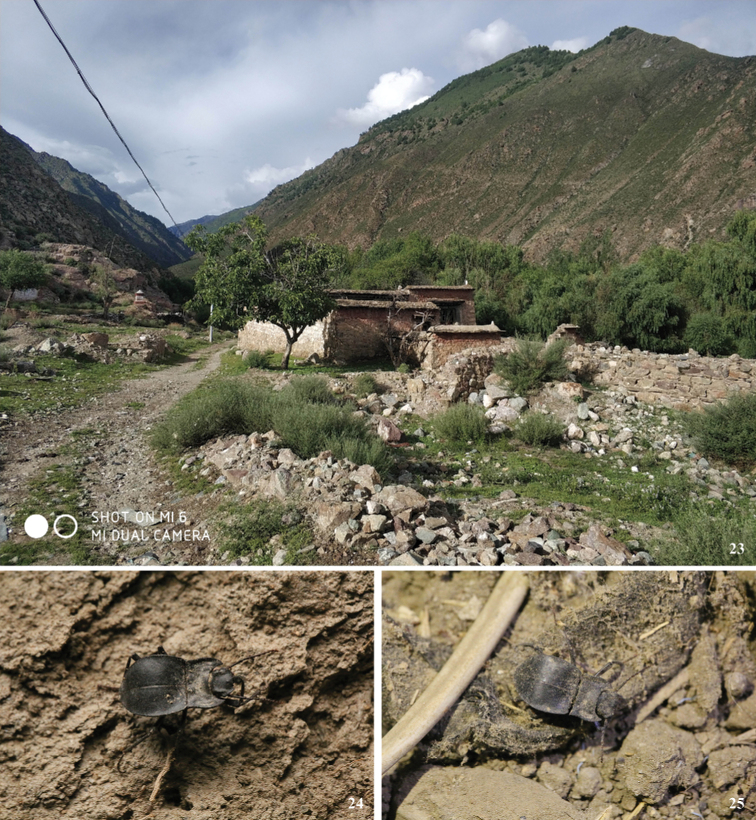
Habitat **23** and adults **24, 25** of *Solskyiainfossata* sp. nov. in Nuqiao Village, Gyaca Town, Gyaca County, Xizang, China (photograph by Zhao Pan).

***Pterothorax*.** Elytra wide-oval, widest near middle, 1.1 times as long as wide; anterior margin nearly straight, base slightly wider than pronotum; lateral sides arcuate, weakly narrowing toward base and strongly narrowing toward apex from middle, lateral margins raised, broad and wrinkled at base; humeri right-angled, rounded apically; surface depressed, with deeper depression at base, strongly convex in middle, declivity sharply sloping downwards; sparsely and finely punctate, shallowly near base, lateral sides and apex, inconspicuously wrinkled; epipleura wide, weakly convex, sparsely and finely punctate, inconspicuously wrinkled. Scutellum triangular.

**Figure 26–29.**
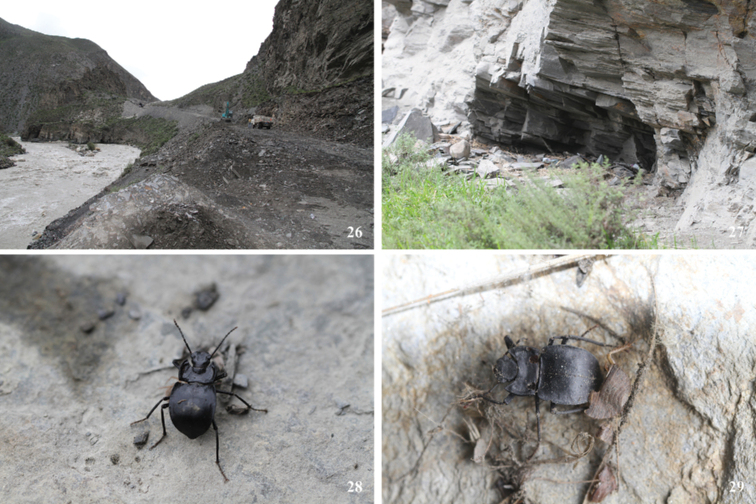
Habitat **26, 27** and adults **28, 29** of *Solskyialhozhaga* sp. nov. in Sênggê Township, Lhozhag County, Xizang, China.

***Abdomen*.** Ventrites strongly convex, densely and coarsely punctate, sparsely and shallowly near lateral sides and apex of the last ventrite; apical margin of the last ventrite widely rounded.

***Legs*.** Slender and long; femora claviform, smooth; tibiae straight, rough; ventral surface of pro- and mesotarsomeres I–IV and metatarsomeres I–III with hairy tuft at apex; claws well developed.

***Aedeagus*.** As in Fig. 10D–F. Length 2.6 mm, width 0.5 mm. Parameres length 2.0 mm, width 0.4 mm.

***Ovipositor*.** As in Fig. 10G, H.

***Sexual dimorphism*.** Females usually with slightly wider and more convex elytra, but in many cases, it is impossible to distinguish the two sexes without extracting the genitalia.

### ﻿ Solskyia parvicollis

Taxon classificationAnimaliaColeopteraTenebrionidae

(Kraatz, 1865)

857B4F43-B8E8-564F-9DA1-5673DBD4E349

Figs 30–33

Akis parvicollis Kraatz, 1865: 251.
Solskyia parvicollis : Gridelli 1934: 51; Español 1961: 127; Ren and Yu 2000: 325 (incorrect spelling as Solskia); Löbl et al. 2008: 127; Iwan et al. 2020: 138.
Cyphogenia plana Bates, 1879: 471.
Solskyia morawitzi Semenov, 1891: 363.
Solskya kuenluna Kaszab, 1965: 282.

#### Material examined.

**China**: 2♂, 3♀ (HBUM), Yecheng County [Kargilik], Xinjiang, 1974-VII-12, leg. Xiang-Chu Yin, Ji-Jun Li; 1♀ (HBUM), Rutog County, Xizang, 1974-VII-11, leg. Xiang-Chu Yin, Ji-Jun Li; 5♂, 7♀ (HBUM), Rutog County, Xizang, 1974-VII-12, leg. Xiang-Chu Yin, Ji-Jun Li; 1♂, 1♀ (HBUM), Rutog County, Xizang, 1974-VII-13, leg. Xiang-Chu Yin, Ji-Jun Li; 7♂, 7♀ (HBUM), Shangqulong, Rutog County, Xizang, 1974-VII-12, leg. Xiang-Chu Yin, Ji-Jun Li; 1 ex. (HBUM), Banggong Co, Rutog County, Xizang, 33°26.714'N, 79°48.618'E, Alt. 4288 m, 2018-VIII-9, leg. Xing-Long Bai, Zhong-Hua Wei, Zi-Yuan Hu, Ming-Min Ma; 30 ex. (HBUM), Wüjang Village, Domar Township, Rutog County, Xizang, 33°37.204'N, 79°49.042'E, Alt. 4311 m, 2018-VIII-9, leg. Xing-Long Bai, Zhong-Hua Wei, Zi-Yuan Hu, Ming-Min Ma; 2♀ (HBUM), Shiquanhe Town, Xizang, 2004-VII-15, leg. Ai-Min Shi, Yi-Bin Ba; 1 ex. (HBUM), Shiquanhe Daban, Gar County, Xizang, 32°19.441'N, 80°00.444'E, 5014 m, 2015-VIII-25, leg. Guo-Dong Ren, Xing-Long Bai, Jun-Sheng Shan; 2 ex. (HBUM), Günsa Township, Gar County, Xizang, 31°54.310'N, 80°06.109'E, 4611 m, 2015-VIII-24, leg. Guo-Dong Ren, Xing-Long Bai, Jun-Sheng Shan; 3 ex. (HBUM), Gê’gyai County, Xizang, 32°23.394'N, 81°09.287'E, 4524 m, 2015-VIII-24, leg. Guo-Dong Ren, Xing-Long Bai, Jun-Sheng Shan.

**Figure 30–33.**
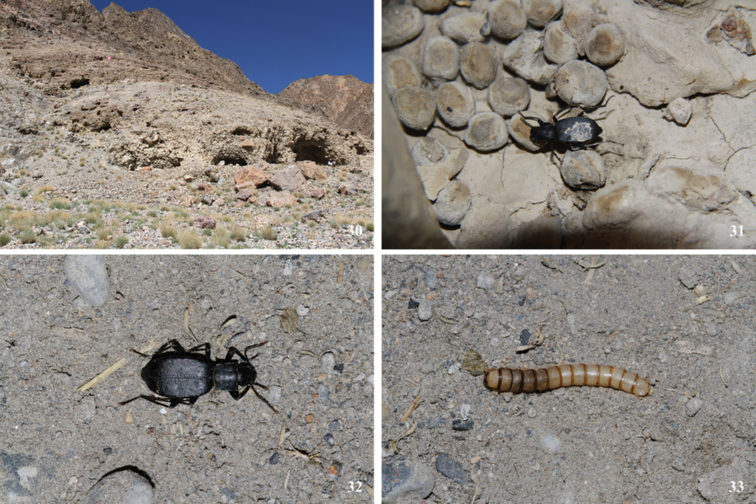
Habitat **30** adults **31, 32** and larva **33** of *Solskyiaparvicollis* (Kraatz) in Wüjang Village, Domar Township, Rutog County, Xizang, China.

#### Distribution.

China: Xinjiang, Xizang; Kashmir, “Himalaya”.

### ﻿Key to known Chinese species of the genus *Solskyia*

**Table:** 

1	Anterior margin of pronotum deeply emarginate, anterior angles sharp and protruding forwards, surface strongly depressed	**2**
–	Anterior margin of pronotum slightly emarginate, anterior angles not sharp and not protruding forwards, surface weakly or not depressed	**4**
2	Body oval elongated; elytra narrow and long, base narrower than pronotum, lateral margins subparallel, humeri widely obtuse-angled, surface of elytra and epipleura with granules	***S.lhasana* Ren & Yu, 2000**
–	Body wide-oval; elytra wide and short, base wider than pronotum, lateral margins arcuate, humeri right-angled, rounded apically, surface of elytra and epipleura with punctures	**3**
3	Lateral margins of pronotum arcuate, posterior angles weakly obtuse	***S.lhozhaga* sp. nov.**
–	Lateral margins of pronotum weakly “S” curved, posterior angles sharp and protruding	***S.infossata* sp. nov.**
4	Posterior angles of pronotum sharp and protruding; humeral carina of elytra elevated, humeri obtuse-angled	***S.caporiaccoi* Gridelli, 1934**
–	Posterior angles of pronotum not sharp and not protruding; humeral carina of elytra inconspicuous at base, humeri rounded	***S.parvicollis* (Kraatz, 1865)**

## Supplementary Material

XML Treatment for Solskyia

XML Treatment for Solskyia caporiaccoi

XML Treatment for Solskyia infossata

XML Treatment for Solskyia lhasana

XML Treatment for Solskyia lhozhaga

XML Treatment for Solskyia parvicollis
